# Genetically determined dietary habits and risk of Alzheimer’s disease: a Mendelian randomization study

**DOI:** 10.3389/fnut.2024.1415555

**Published:** 2024-06-03

**Authors:** Fei Teng, Jiahui Sun, Zheyu Chen, Hao Li

**Affiliations:** ^1^Department of Liver Surgery, West China Hospital of Sichuan University, Chengdu, China; ^2^Wangjing Hospital of China Academy of Chinese Medicine Sciences, Beijing, China

**Keywords:** Alzheimer’s disease, diet, dietary habits, Mendelian randomization study, oily fish consumption

## Abstract

**Background:**

Emerging evidence have suggested that dietary habits have potential implication on the development of Alzheimer’s disease (AD). However, elucidating the causal relationship between specific dietary factors and AD risk remains a challenge. Therefore, our study endeavors to investigate the causal association between dietary habits and the risk of AD.

**Materials and methods:**

We analyzed data on 231 dietary habits sourced from the UK Biobank and MRC-IEU, and AD data obtained from the FinnGen database. Employing a framework based on the classic two-sample Mendelian randomization (MR) study, we utilized the inverse-variance weighted (IVW) method as the primary analysis. Additionally, we conducted Steiger filtering and other methods to mitigate horizontal pleiotropy. The robustness of our overall findings was confirmed through multiple sensitivity analysis methods, and forward MR and reverse MR to address potential reverse causality bias.

**Results:**

Our study evaluated the causal effect between 231 dietary habits involving over 500,000 participants of European ancestry, and 10,520 AD cases. Only oily fish intake demonstrated a significant protective causal relationship with AD following FDR correction (raw *p*-value = 1.28e-4, FDR *p*-value = 0.011, OR = 0.60, 95%CI: 0.47–0.78). Additionally, six dietary habits potentially influenced AD risk, with protective causal effects observed for average monthly intake of other alcoholic drinks (raw *p*-value = 0.024, FDR *p*-value = 0.574, OR = 0.57, 95%CI: 0.35–0.93) and tea intake (raw *p*-value = 0.047, FDR *p*-value = 0.581, OR = 0.78, 95%CI: 0.603–1.00). Conversely, detrimental causal effects were observed for the average weekly champagne plus white wine intake (raw *p*-value = 0.006, FDR *p*-value = 0.243, OR = 2.96, 95%CI: 1.37–6.38), Danish pastry intake (raw *p*-value = 0.036, FDR *p*-value = 0.574, OR = 13.33, 95%CI: 1.19–149.69), and doughnut intake (raw *p*-value = 0.039, FDR *p*-value = 0.574, OR = 7.41, 95%CI: 1.11–49.57). Moreover, the protective effect of goat’s cheese intake phenotype exhibited statistical significance only in the IVW method (raw *p*-value<0.05).

**Conclusion:**

Our results provide genetic support for a protective causal effect of oily fish intake on AD risk. Additionally, average monthly intake of other alcoholic drinks and tea consumption were also related with a lower risk of AD. Conversely, average weekly champagne plus white wine intake, Danish pastry intake, and doughnut intake were causally associated with increased risk of AD.

## 1 Introduction

Alzheimer’s Disease (AD) is a progressive neurodegenerative disorder characterized by cognitive decline, memory impairment, and functional deficits, posing a pressing global health challenge (1–3). Despite extensive research, effective treatments for AD remain elusive, underscoring the urgency to identify modifiable risk factors and preventive strategies (4, 5).

In the realm of AD research, dietary habits have garnered considerable attention due to emerging evidence suggesting their potential implication in disease pathogenesis (6–9). However, the causal relationship between dietary factors and AD risk remains inadequately understood, partly due to inherent limitations in traditional observational studies, such as confounding and reverse causation. There were several observational studies and previous Mendelian randomization (MR) analyses reported associations between specific dietary components (such as coffee, micronutrients, lipids, and salt intake) and AD risk (10–14). Furthermore, our study aims to address the gaps and inconsistencies in the literature pertaining to the association between dietary habits and AD risk.

MR analysis provides a robust method to elucidate causal relationships between exposures and outcomes by leveraging genetic variation as instrumental variables (IVs), overcoming inherent limitations in traditional observational studies (15, 16). MR analysis offers more reliable evidence concerning the potential impact of dietary habits on AD risk.

We adopt a classic two-sample MR approach to probe potential causal links between varied dietary habits and AD risk, with implications for preventive interventions, dietary guidelines, and targeted strategies aimed at alleviating the burden of AD on individuals and society as a whole. Sensitivity analyses are conducted for all MR analysis results. To mitigate the risk of reverse causality bias, we implement both forward MR and reverse MR methods.

## 2 Methods and materials

### 2.1 Study design

Our study employed a framework based on the classic two-sample MR study to assess the potential causal relationship between different dietary habits (exposures) and AD (outcome) (17, 18). MR offers advantages over retrospective studies by mitigating numerous bias factors. However, its analysis results hinge on the comprehensive application of three fundamental principles: strong correlation between IVs and exposure factors, absence of significant association between IVs and confounding factors, and no direct impact of IVs on outcome factors.

**Figure 1:**
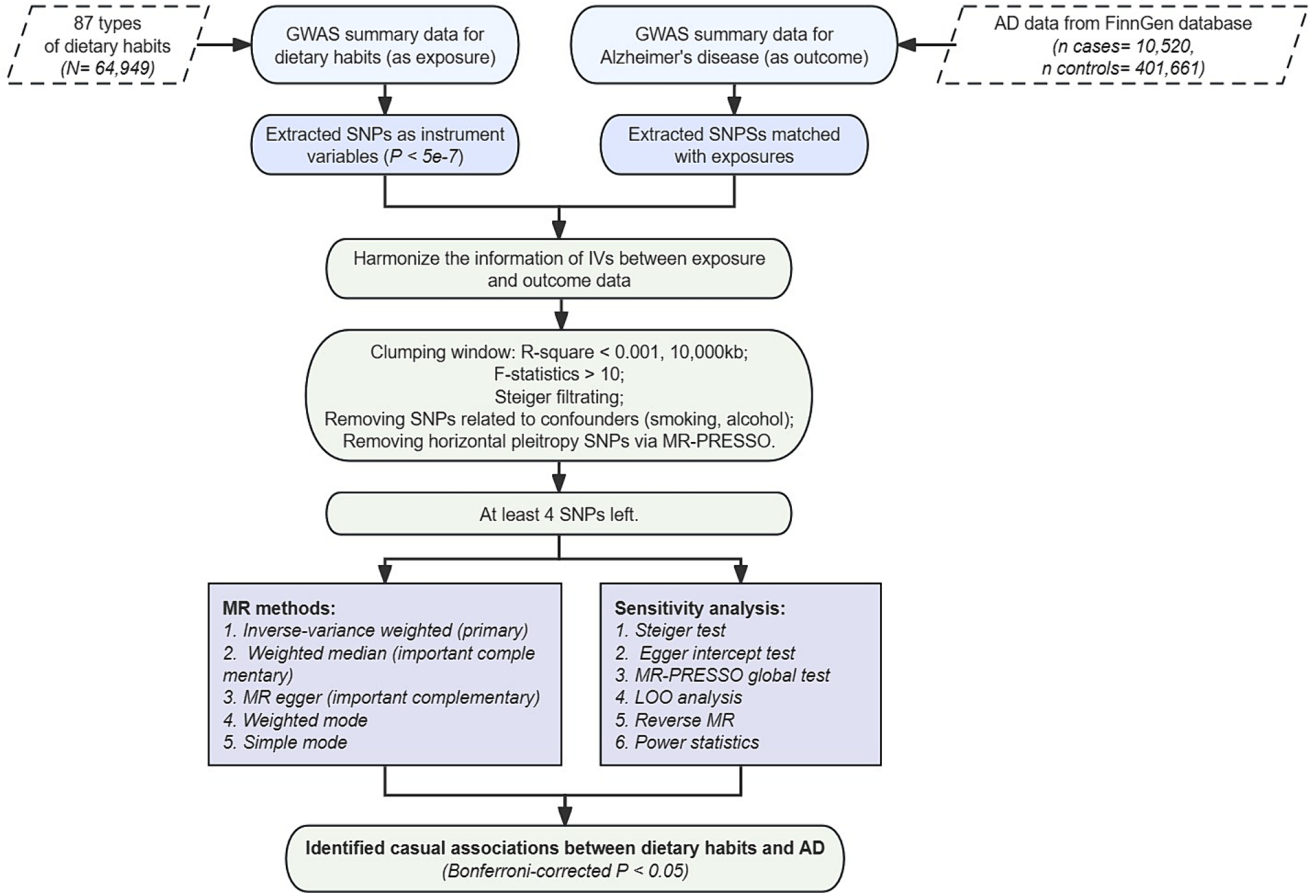
Flow chart of study design.

To uphold these principles, our study design integrated various methodologies. We ensured the efficacy of IVs through *p*-value filtering, F-statistic calculation, and linkage disequilibrium (LD) filtering. Additionally, techniques such as Steiger filtering, MR-PRESSO filtering, and exclusion of SNPs associated with confounding factors mitigated horizontal pleiotropy. The stability of our overall test was affirmed through computations including Egger intercept, leave-one-out (LOO), and multiple sensitivity analysis methods. Ultimately, reverse confounding bias was addressed through reverse MR analysis and the Steiger test, as depicted in Figure 1.

### 2.2 Data sources

The exposure factor dataset was sourced from the UK Biobank’s dietary questionnaire data and summary-level genome-wide association study (GWAS) data processed by MRC-IEU (19, 20). With over 500,000 participants of European ancestry, the UK Biobank accommodated a broad spectrum of dietary habits, encompassing 231 analyzed by MRC-IEU. Post-SNP quality control and minor allele frequency (MAF) filtering, MRC-IEU conducted multiple regression analyses, incorporating dietary habit intake as a variable alongside 10 principal components, to compute the effects and *p*-values of all SNPs on the unit standard deviations (SD) effect size. Detailed data control reports, along with summary-level GWAS data for all exposure phenotypes, are accessible via OpenGWAS,^1^ with comprehensive exposure data IDs and corresponding phenotypes provided in Supplementary materials.

Outcome data were derived from version R10 of the FinnGen database’s summary-level GWAS data for AD disease (21), encompassing 10,520 cases and 401,661 unaffected control subjects of primarily European ancestry. Following stringent SNP filtering and quality control processing, linear regression was executed, incorporating the top 10 principal components, to ascertain the effect of each SNP on the unit logOR value and the corresponding *p*-value.

### 2.3 Genetic instruments identification

To ensure the reliability of incorporating phenotypes and SNPs as IVs, we implemented stringent criteria. Initially, we restricted the set of SNPs to be directly associated with the exposure at the genome-wide significant *p*-value threshold at *p* < 5e-7 (22). Furthermore, phenotypes meeting this threshold were cross-referenced with SNP data from the 1,000 Genomes Project European ancestry cohort to calculate SNP intervals and query MAF. Ambiguous SNPs with unclear sequences and those containing palindromic sequences were eliminated, filtering out rare variant SNPs with MAF < 0.1. Subsequently, to mitigate the impact of LD on the independence of IVs, we set the SNP clustering distance window to 10,000 kb and computed the LD independence correlation *r*^2^, thereby removing SNPs with *r*^2^ < 0.01.

To mitigate bias from weak IVs, we concurrently calculated the F-statistic (23) to evaluate their strength as IVs, subsequently discarding weak instrumental SNPs with *F* values <10. Following this, we utilized the MR-PRESSO package (24) to iteratively test and exclude SNPs exhibiting horizontal pleiotropy, and applied Steiger filtering (25) to eliminate SNPs more strongly associated with the outcome factor than the exposure factor, thereby further mitigating reverse causality at the SNP level.

Finally, we excluded SNPs highly associated with smoking, alcohol consumption, and type 2 diabetes through the GWAS catalog database. Only exposure phenotype traits with a remaining SNP count greater than 4 were included for subsequent analysis, ensuring the reliability and validity of our study.

### 2.4 Mendelian randomization analysis

Our study employed five different MR methods, cross-referencing multiple approaches to enhance result credibility. Among these, we utilized the IVW method (26), extensively validated for its robustness, as the primary MR outcome. The IVW method conducts meta-analysis of Wald values to determine the final causal effect, serving as an accurate assessment of statistical efficacy when other methods conflict in terms of statistical significance.

Additionally, we introduced Weighted Median (27) and MR-Egger methods (28) as primary references for the results. These methods, compared to the IVW method, allow greater pleiotropy and presence of invalid SNPs but tend to be conservative in result assessment. The Weighted Median method permits up to half the SNPs exhibiting pleiotropy, while the MR-Egger method remains applicable even when all SNPs may be invalid. We considered MR results robust only when the Weighted Median and MR-Egger methods demonstrated consistent causal effects with the IVW method, and at least the IVW method indicated statistical differences (*p*-value threshold of 0.05) without apparent pleiotropy or reverse causality. Furthermore, we included Weighted Mode and Simple Mode as supplementary MR methods for comparison with the aforementioned three methods. The MR results were deemed robust when all five methods exhibited consistent effect directions.

In the forward MR analysis, each dietary phenotype was considered as exposure, with AD data from the FinnGen database as the outcome, to complete the two-sample MR. To exclude reverse causality, in the reverse MR stage, AD data from different databases were considered as exposure, with multi-omics phenotypes as outcomes to evaluate their causal effects. The criteria for extracting IVs and selecting MR methods were consistent with the forward MR stage. However, SNPs involved in the forward two-sample stage were not utilized for reverse testing. Additionally, to enhance the reliability of excluding reverse causal associations, we employed the Steiger test, considering a *p*-value <0.05 indicative of the absence of reverse causality in the MR analysis.

### 2.5 Sensitivity and heterogeneity

In light of the fundamental assumption that underpins the robustness of the IVW method, which hinges on the absence of apparent pleiotropy, our study employed three sensitivity analysis techniques, namely the Egger intercept, MR-PRESSO global *p*-value, and leave-one-out (LOO) analysis (29). Among these, the MR-Egger method facilitates the computation of the intercept value in causal LASSO regression and ascertains the statistical effect associated with this intercept value. A *p*-value below 0.05 for the intercept value denotes a substantial risk of potential pleiotropy. In contrast to MR-Egger, MR PRESSO exhibits superior capability in accurately appraising the presence of potential pleiotropy, demonstrating heightened sensitivity to its existence. Likewise, MR PRESSO is adept at gauging the presence of global pleiotropy, signifying its occurrence when the *p*-value falls below 0.05.

Moreover, we conducted an assessment of the impact on MR by systematically eliminating each SNP to mitigate the influence of outlier SNPs, thereby diminishing pleiotropy and heterogeneity. A LOO forest plot was generated to visually present the final outcomes. Reliability in IVW results is contingent upon both the Egger intercept and MR-PRESSO corroborating the absence of significant evidence of pleiotropy, and the MR analysis remaining unaffected by individual SNPs. Additionally, heterogeneity was appraised through Cochran’s Q test (30) for both IVW and MR-Egger methods, in conjunction with funnel plots. A *p*-value below 0.05 in Cochran’s Q test signifies the presence of heterogeneity, warranting a cautious interpretation of the MR results in instances of instability. Funnel plots offer a subjective assessment of the extent of heterogeneity.

### 2.6 Power statistics calculation

In addition to sensitivity and heterogeneity analyses, we adopted a pioneering method for computing statistical power in MR analysis.^2^ This tool employs asymptotic theory estimation to assess the reliability of causal effects deduced from MR. Power calculations were executed at a Type I error rate of 0.05, factoring in parameters such as *r*^2^, the extent of GWAS data, and the OR derived from MR analysis utilizing the IVW method.

### 2.7 Statistics

The foundation for all analysis conducted was the R software platform (version 4.3.1). Bidirectional MR studies utilized the “TwoSampleMR” software package, supported by the MR-PRESSO method provided by the “MR-PRESSO” software package. We set a Type I error acceptance threshold of 0.05, whereby original *p*-values obtained from the IVW method underwent FDR correction. Corrected *p*-values <0.05 were deemed indicative of significant causal relationships. Results with an uncorrected *p*-value <0.05 but a corrected *p*-value >0.05 were interpreted as potentially suggestive of underlying causal relationships, with causal effect estimates presented as OR and accompanied by 95% CI.

## 3 Results

### 3.1 Genetic IVs

From the dietary-related questionnaire GWAS data of the UK Biobank, we extracted a total of 1,768 SNPs meeting the correlation threshold of *p* < 5e-7. All SNPs across phenotypes underwent Steiger testing to exclude weak IVs and outliers (MR-PRESSO outlier test, *p* < 0.05). Ultimately, 87 dietary phenotype traits with at least four SNPs were included for subsequent analysis. All phenotypes were categorical variables, with units expressed in SD. The detailed information of the selected IVs for subsequent MR analysis is provided in Supplementary Table S2.

### 3.2 Main MR results

The results of the MR analysis revealed that, subsequent to FDR correction of IVW method-derived *p*-values, only the oily fish intake phenotype (raw *p*-value = 1.28e–4, FDR *p*-value = 0.011, OR = 0.60, 95%CI: 0.47–0.78) demonstrated a significant protective causal relationship with AD. OR values obtained from the other four MR statistical methods were uniformly less than 1, indicating consistent directional outcomes. Notably, the Weighted median method, serving as a crucial supplement, exhibited statistically significant disparities akin to the IVW method (*p*-value = 0.010, OR = 0.60, 95%CI: 0.41–0.88), while the MR Egger method adopted a more conservative stance compared to the Weighted median method, yielding a non-significant *p*-value.

Moreover, six dietary habit phenotypes potentially exerting causal effects on AD were identified. Among these, dietary habit phenotypes potentially conferring protective causal effects on AD encompass: average monthly intake of other alcoholic drinks (raw *p*-value = 0.024, FDR *p*-value = 0.574, OR = 0.57, 95%CI: 0.35–0.93), tea intake (raw *p*-value = 0.047, FDR *p*-value = 0.581, OR = 0.78, 95%CI: 0.603–1.00); while those manifesting potentially pathogenic causal effects on AD include: average weekly champagne plus white wine intake (raw *p*-value = 0.006, FDR *p*-value = 0.243, OR = 2.96, 95%CI: 1.37–6.38), Danish pastry intake (raw *p*-value = 0.036, FDR *p*-value = 0.574, OR = 13.33, 95%CI: 1.19–149.69), doughnut intake (raw *p*-value = 0.039, FDR *p*-value = 0.574, OR = 7.41, 95%CI: 1.11–49.57). However, although the goat’s cheese intake phenotype exhibited statistical significance in the IVW method of MR analysis concerning AD (raw *p*-value <0.05), its result lacked robustness and was excluded due to the disparate effect direction (OR > 1) obtained by the MR Egger method in contrast to the other four methods (OR < 1).

**Figure 2:**
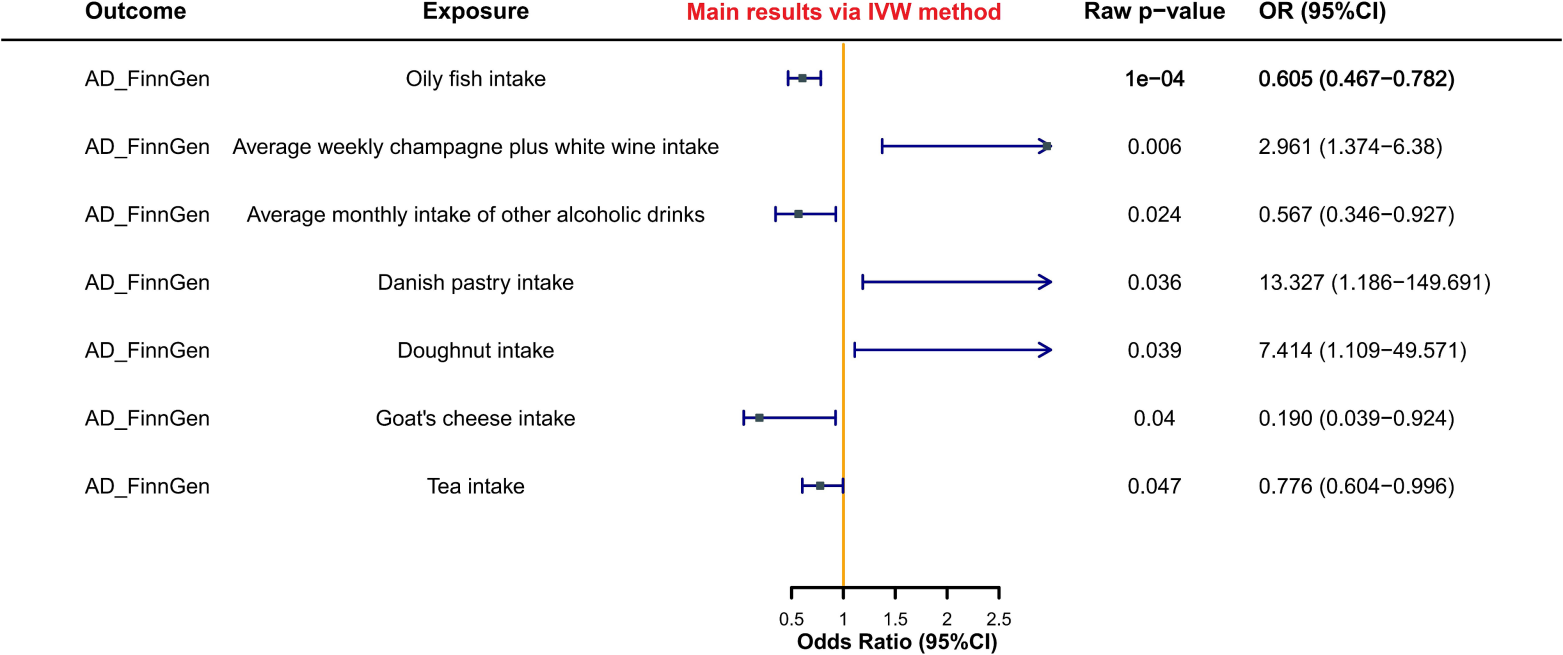
Forest plot of MR study between dietary habits and AD.

Figure 2 illustrated specific results, while Supplementary Table S1 provided a summary of all phenotypes jointly analyzed with AD. Detailed analyses were available in Supplementary File 2.

### 3.3 Sensitivity and heterogeneity

The findings indicated that the 6 dietary habit phenotypes previously identified as having causal or potential causal relationships with AD passed all three sensitivity methods. This suggested the absence of significant pleiotropy and influential outliers. Moreover, their statistical power calculations ranged from 0.9 to 1, indicating the capacity to effectively mitigate type II statistical errors. However, both the Vegetarian sausages/burgers intake and Average weekly beer plus cider intake phenotypes exhibited statistically significant intercept values (*p* < 0.05) in the Egger intercept method. Similarly, the poultry intake, average weekly red wine intake, bread intake, cheese intake, and cereal intake phenotypes demonstrated statistical significance (*p* < 0.05) in MR-PRESSO global sensitivity *p*-values, despite outlier removal failing to impact their sensitivity statistical significance. Thus, it remains inconclusive whether these phenotypes have potential causal relationships with AD.

Concerning heterogeneity, the primary dietary habit phenotypes with significant causal or potential causal relationships with AD displayed significant heterogeneity in the Q-statistic calculated by both the IVW and MR-Egger methods. This underscores the consistency among IVs and the robustness of the results. Detailed LOO plots for each analysis were provided in Supplementary File 2. For Egger intercept, MR-PRESSO global sensitivity *p*-values, and heterogeneity analysis results, please consult Supplementary Table S1.

### 3.4 Reverse MR analysis

Our findings indicated that the Steiger test *p*-values associated with the 6 principal dietary habit phenotypes, which exhibited significant or potential causal relationships with AD, were markedly below the threshold of 0.05. This underscores the statistical significance of the *p*-values pertaining to the accurate direction of MR analysis. Moreover, the IVW method employed in reverse MR analyses yielded no statistically significant reverse causal associations (*p* > 0.05).

## 4 Discussion

Our study conducted a two-sample MR analysis to evaluated the causal effect between 231 dietary habits, involving over 500,000 participants of European ancestry, and 10,520 AD cases. Among these, only oily fish intake demonstrated a significant protective causal relationship with AD following FDR correction (raw *p*-value = 1.28e-4, FDR *p*-value = 0.011, OR = 0.60, 95%CI: 0.47–0.78). Additionally, 6 dietary habits potentially influenced AD risk, with protective effects observed for average monthly intake of other alcoholic drinks and tea intake, and detrimental effects for average weekly champagne plus white wine intake, Danish pastry intake, and doughnut intake. However, goat’s cheese intake lacked robustness and was excluded due to disparate effect directions.

A handful of observational studies have investigated the association between oily fish intake and AD, but the studies found that fatty fish consumption was associated with a reduced risk of AD (31, 32). Moreover, evidence from a meta-analysis of prospective cohort studies revealed that while increased fish intake was associated with a decreased risk of AD (33), which was consistent with our findings. Epidemiological evidence consistently linked consumption of omega-3 fatty acids, especially docosahexaenoic acid (DHA), from fatty fish and fish oils with cognitive benefits across various age groups (34, 35). Despite promising epidemiological findings, randomized controlled trials (RCTs) evaluated the effects of omega-3 fatty acid supplementation, specifically eicosapentaenoic acid (EPA) and DHA, might lower the risk of cognitive decline and dementia (36–38). Mechanistic insights from experimental models suggested a potential role for omega-3 fatty acids, particularly DHA and EPA, in mitigating neuroinflammation, a process implicated in AD pathogenesis (39, 40). In addition, the potential influence of APOE genotype, particularly APOE4 carriers who were more contribute to AD, were more beneficial to omega-3 fatty acid supplementation (41). However, future studies should be performd to clarify the underlying mechanistic pathways between oily fish intake and AD.

A comprehensive examination of the relationship between tea intake and AD risk revealed multifaceted findings. Evidence from a systematic review of observational studies indicated that green tea intake might reduce AD risk (42). Moreover, a meta-analysis of prospective studies revealed that the tea could be inversely associated with neurodegenerative disorders (43), which was also consistent with our results. Conversely, a MR study genetically suggested a potential causal link between increased tea intake and elevated AD risk, with genetically predicted tea intake associated with decreased brain volume, particularly in the gray matter and right hippocampus (44). Finally, a dose–response meta-analysis indicated an inverse association between tea consumption and cognitive disorders, suggesting 1 cup/day of tea intake leads to an 11% reduction in cognitive deficits (45). Overall, while tea consumption offers some cognitive health benefits, its nuanced effects on AD risk warrant further investigation. Furthermore, research highlights the neuroprotective potential of methylxanthines (46) and flavonoid intake (47), prevalent in tea, against neurodegenerative diseases like AD. Conversely, analysis of trace metals in tea samples indicates negligible risks of fluorosis and AD associated with tea consumption, though elevated carcinogenic risk levels for arsenic warrant attention (48).

As for alcoholic drinks, the literature on alcohol intake and AD risk presents diverse findings, highlighting both potential benefits and risks associated with alcohol consumption. Studies indicated that a polyphenols-enriched diet might confer cognitive benefits and attenuate aspects of the neuropathological cascade in AD mouse models (49). Moreover, research revealed that the role of oral and intestinal microbiota in mediating the effects of wine polyphenols on AD pathology, emphasizing the interplay between microbial metabolites and brain communication pathways (50). Average monthly intake of other alcoholic drinks decreased AD risk might be benefit from enriched polyphenols. However, conflicting evidence exists regarding the relationship between alcohol intake and AD risk. While some studies suggest that moderate consumption of wine may improve cognitive performance and mitigate AD risk (51). Average weekly champagne plus white wine intake increased AD risk might associated with excessive alcohol consumption. Because champagne was a kind of sparkling wine, building upon the observation that carbonated alcoholic beverages can expedite the absorption rate of ethanol, it is hypothesized that carbon dioxide (CO2) molecules may similarly enhance the absorption of alcohol.

As we all know, Danish pastry and doughnut were high-fat diet. Recent studies have focused on the association between high-fat diet (HFD) and AD risk. Research indicated that HFD-induced prediabetes had a more pronounced impact on females, exacerbating cognitive deficits and neuropathological changes in AD (52). The mechanism between HFD and AD pathogenesis might be related to inflammation-driven pathways, like C/EBPβ/AEP signaling (53). Additionally, obesity-associated immune dysregulation accelerates recognition-memory impairment in AD models, highlighting the crosstalk between metabolic dysfunction, immune activation, and cognitive decline in AD (54).

The study has several strengths. Firstly, our study employed a rigorous MR approach to investigate the potential causal links between varied dietary habits and AD risk. Three MR model assumptions were met by a series of methodology. Our study conducted stringent *p*-value thresholds, F-statistic evaluation, and LD filtering to ensure the efficacy of IVs. Steiger and MR-PRESSO filtering mitigated potential issues of horizontal pleiotropy and reverse confounding bias. Sensitivity analyses and power calculations assessed result robustness and reliability. Secondly, the study population was limited to individuals of European ancestry, thereby reducing potential bias stemming from population stratification.

The present MR study has several limitations. First, the LOO experiment was conducted to assess the impact of excluding each SNP, but the small number of remaining SNPs restricted our ability to support multiple SNP sets. Second, regarding database replacement, challenges arise due to the requirement for different populations in the exposure and outcome data sources while maintaining a consistent genetic background. Although the FinnGen dataset offers a sizable sample size and relevant genetic background, accessing comparable data from other non-UK populations with European ancestry and sufficient sample size proves difficult. While acknowledging the necessity of validating our findings using larger non-UK datasets or alternative research methods in the future, we remain cautious in drawing conclusions by comparing them with existing literature. Third, phenotypic data derived from self-reported dietary questionnaires might be subject to recall bias and measurement error. Forth, although our MR study conducted a rigorous approach to mitigate potential horizontal pleiotropy and confounding bias, it still exits several potential risk unmeasured confounding factors. This limitation derived from MR analysis itself. Lastly, the utilization of GWAS data from European ancestry limitated the generalizability of the findings to the wider population.

Future research should explore potential biological mechanisms underlying the observed associations and investigate the impact of dietary interventions on AD risk. Additionally, longitudinal studies are warranted to assess the long-term effects of dietary habits on AD development. Furthermore, incorporating diverse populations and exploring gene–environment interactions may provide further insights into the relationship between diet and AD.

## 5 Conclusion

In conclusion, our study provides supportive evidence for genetic variation of oily fish intake was associated with a lower risk of AD. Additionally, average monthly intake of other alcoholic drinks and tea intake was also associated with decreased risk of AD. However, our results suggest average weekly champagne plus white wine intake, Danish pastry intake, and doughnut intake had negative causal directions with AD risk. Ultimately, a better understanding of the relationship between dietary habits and AD risk may pave the way for targeted dietary interventions to mitigate the burden of this debilitating neurodegenerative disease.

## Data availability statement

The original contributions presented in the study are included in the article/Supplementary material, further inquiries can be directed to the corresponding authors.

## Author contributions

FT: Conceptualization, Data curation, Formal analysis, Investigation, Methodology, Software, Visualization, Writing – original draft. JS: Conceptualization, Data curation, Formal analysis, Investigation, Methodology, Software, Validation, Visualization, Writing – original draft. ZC: Project administration, Resources, Supervision, Writing – review & editing. HL: Funding acquisition, Project administration, Resources, Supervision, Writing – review & editing.

## Footnotes

^1^ https://gwas.mrcieu.ac.uk

^2^ https://shiny.cnsgenomics.com/mRnd/
