# Supplementary figures and images for: Genetically determined dietary habits and risk of Alzheimer’s disease: a Mendelian randomization study
Source: Front Nutr. 2024 Jun 3;11:1415555. doi: 10.3389/fnut.2024.1415555 (PMC11180739; doi:10.3389/fnut.2024.1415555)

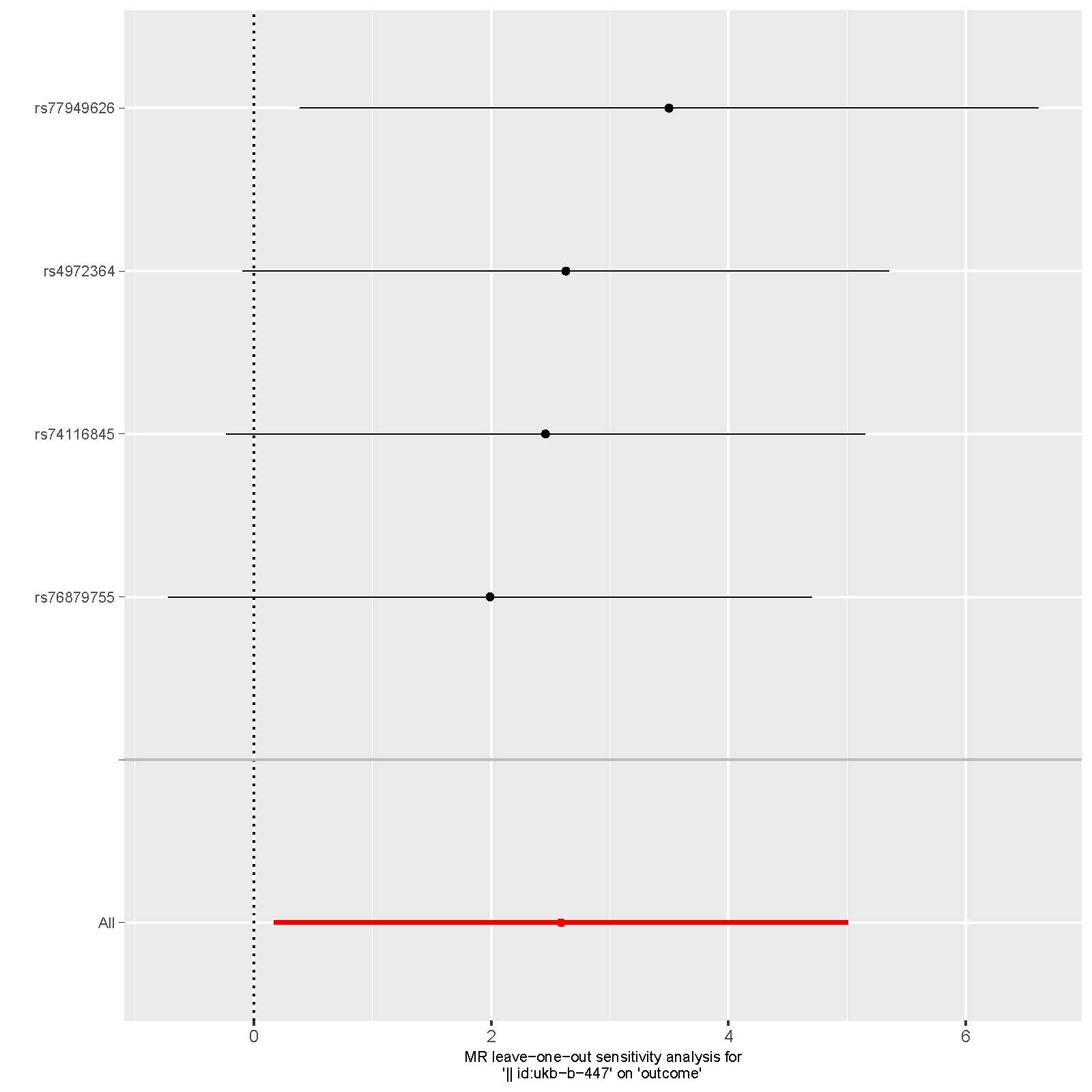

Supplement: Supplementary file 4 [file Image_1.JPEG]

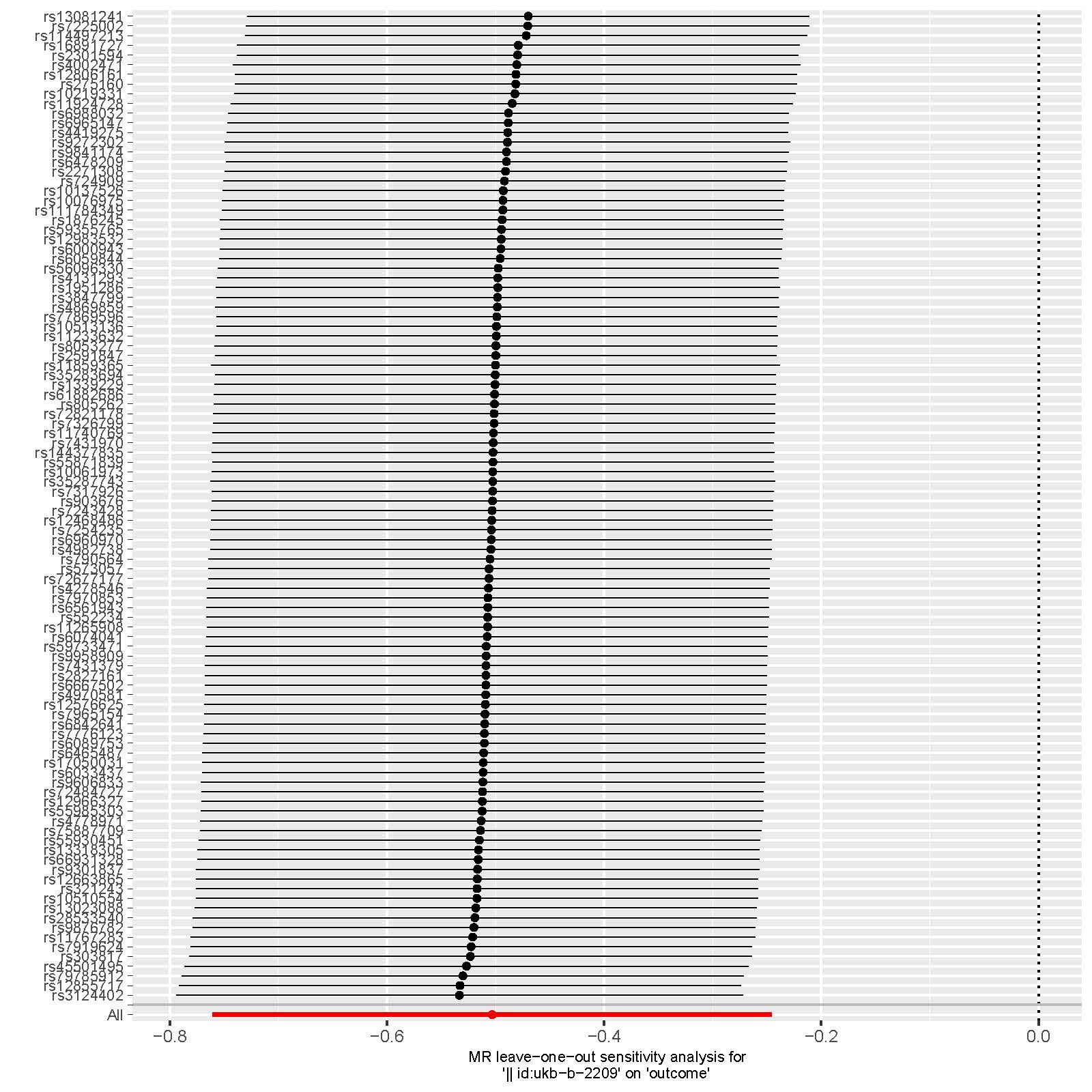

Supplement: Supplementary file 5 [file Image_2.JPEG]

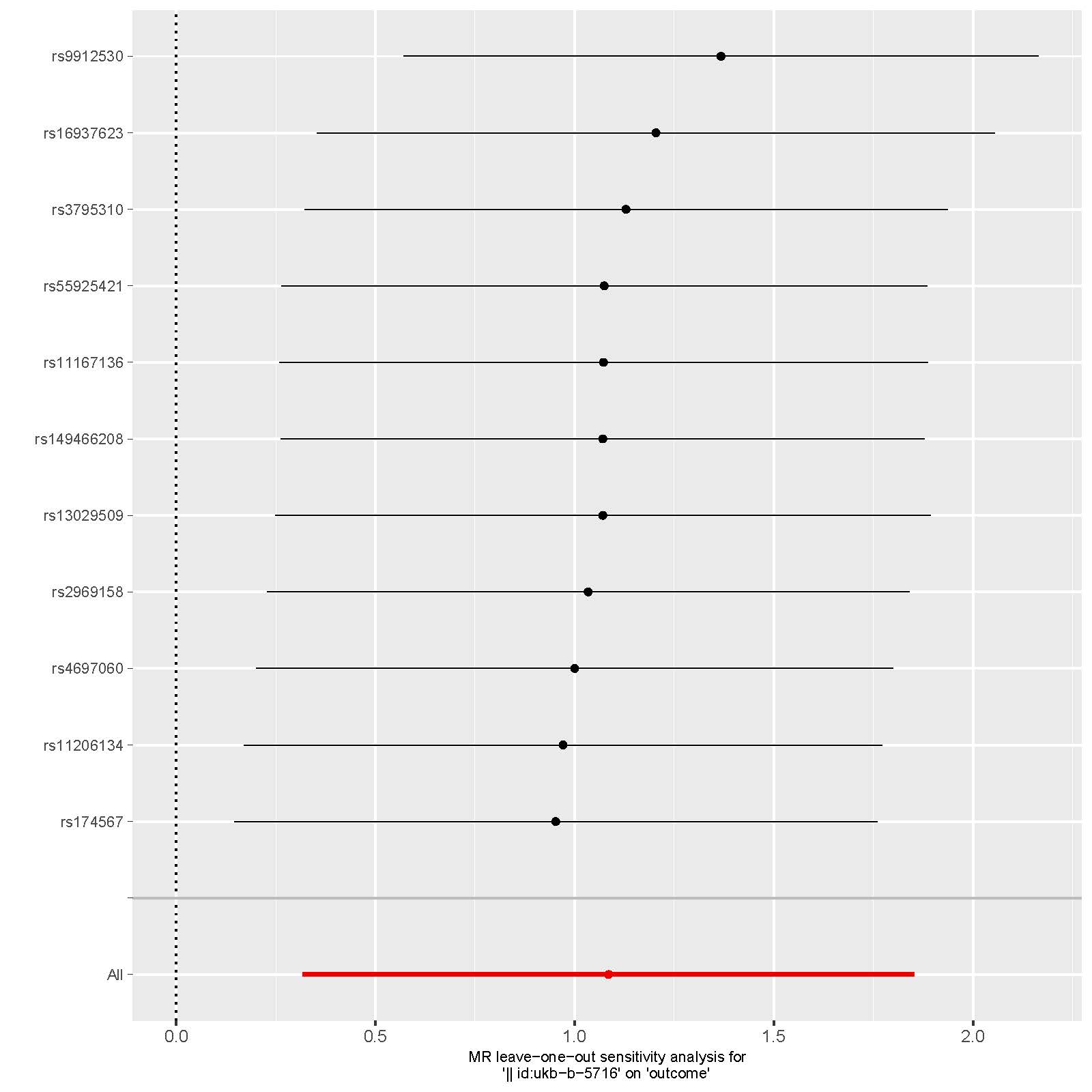

Supplement: Supplementary file 6 [file Image_3.JPEG]

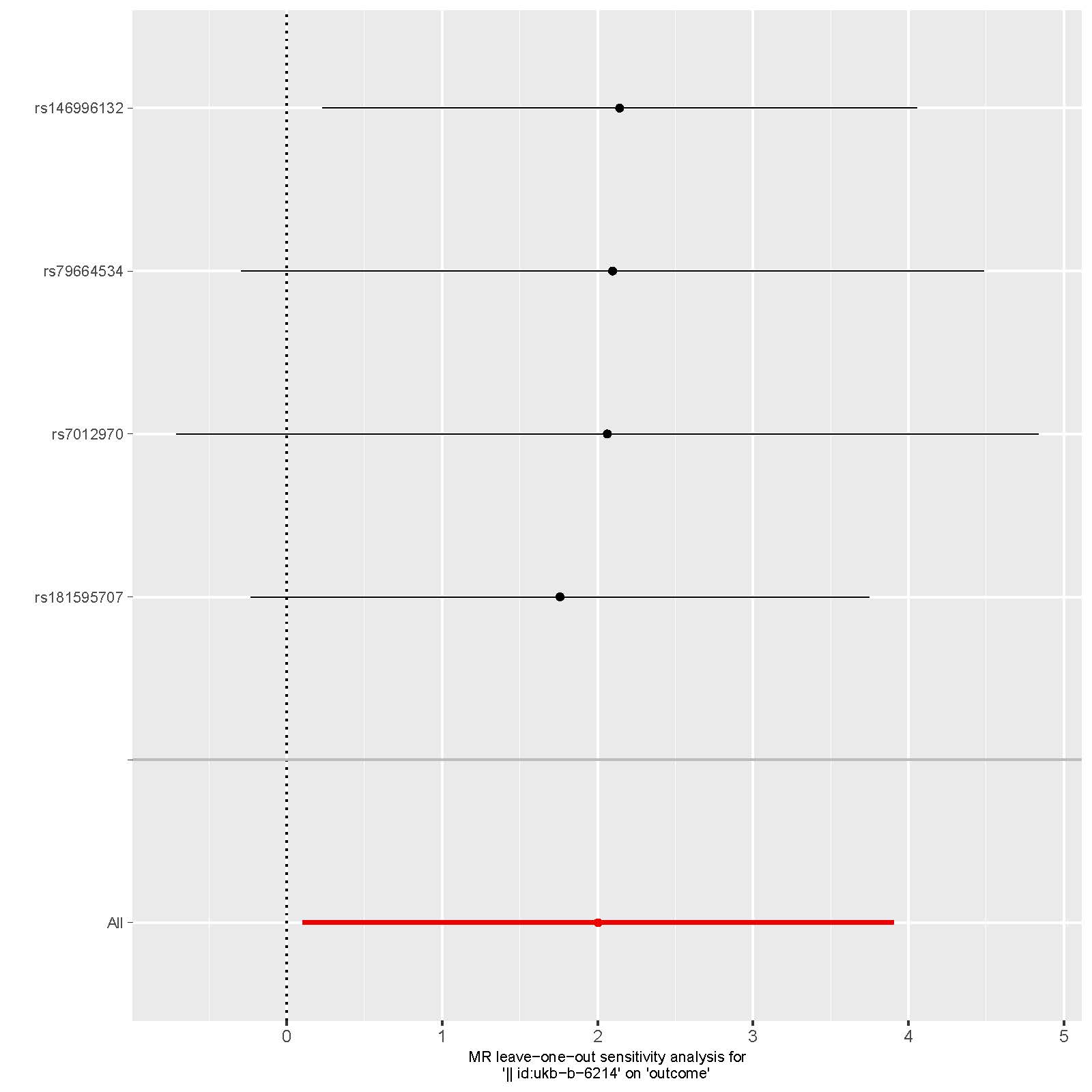

Supplement: Supplementary file 7 [file Image_4.JPEG]

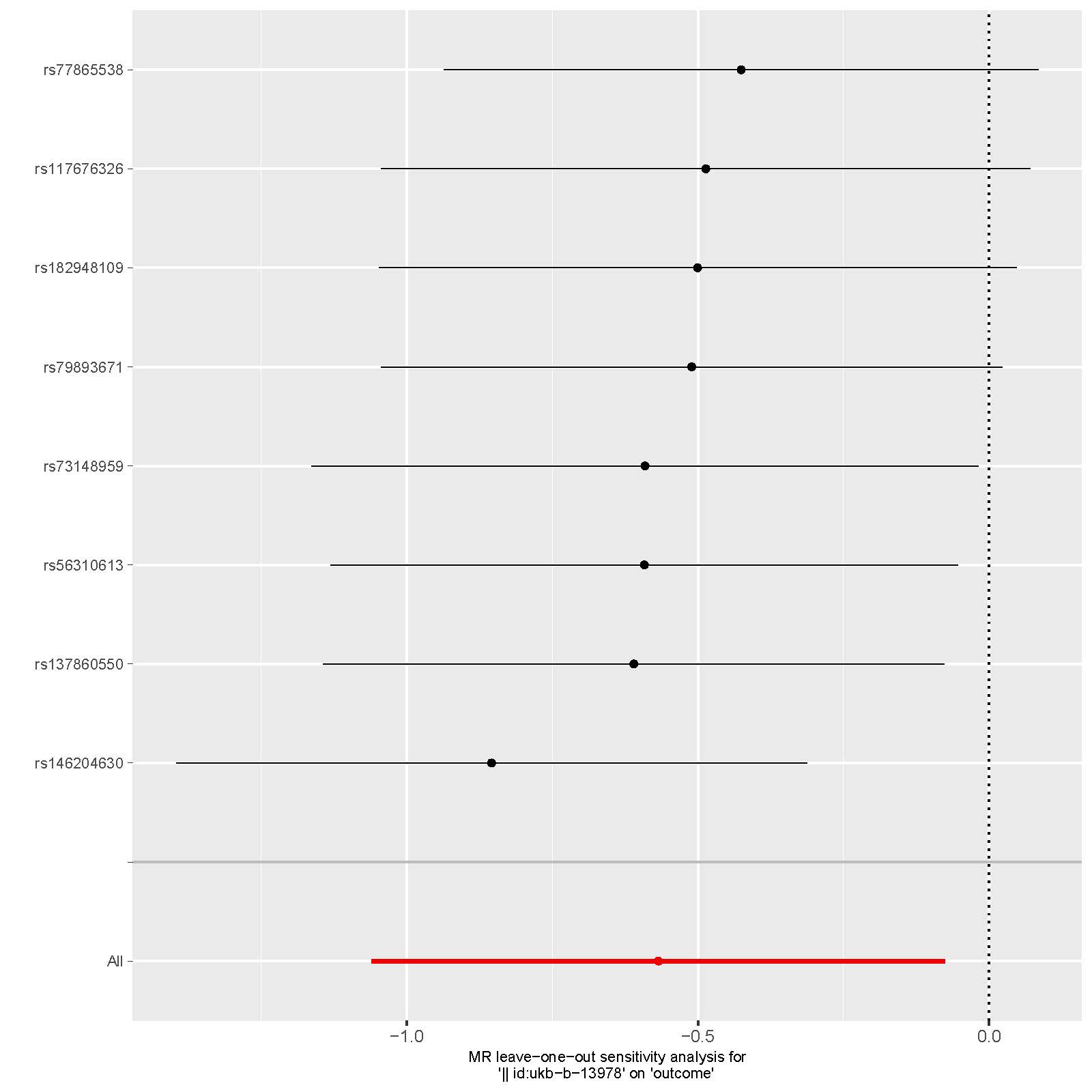

Supplement: Supplementary file 8 [file Image_5.JPEG]
